# The link between transcript regulation and de novo protein synthesis in the retrograde high light acclimation response of Arabidopsis thaliana

**DOI:** 10.1186/1471-2164-15-320

**Published:** 2014-04-30

**Authors:** Marie-Luise Oelze, Meenakumari Muthuramalingam, Marc Oliver Vogel, Karl-Josef Dietz

**Affiliations:** Biochemistry and Physiology of Plants, Faculty of Biology – W5-134, University of Bielefeld, 33501 Bielefeld, Germany

## Abstract

**Background:**

Efficient light acclimation of photosynthetic cells is a basic and important property of plants. The process of acclimation depends on transformation of retrograde signals in gene expression, transcript accumulation and *de novo* protein synthesis. While signalling cues, transcriptomes and some involved players have been characterized, an integrated view is only slowly emerging, and information on the translational level is missing. Transfer of low (8 μmol quanta^.^m^-2.^s^-1^) or normal light (80 μmol quanta^.^m^-2.^s^-1^) acclimated 30 d old *Arabidopsis thaliana* plants to high light (800 μmol quanta^.^m^-2.^s^-1^) triggers retrograde signals. Using this established approach, we sought to link transcriptome data with *de novo* synthesized proteins by *in vivo* labelling with ^35^S methionine and proteome composition.

**Results:**

De novo synthesized protein and proteome patterns could reliably be matched with newly annotated master gels. Each molecular level could be quantified for a set of 41 proteins. Among the proteins preferentially synthesized in plants transferred to high light were enzymes including carbonic anhydrase, fructose-1,6-bisphosphate aldolase, O-acetyl serine thiol lyase, and chaperones, while low rates upon transfer to high light were measured for e.g. dehydroascorbate reductase, glyceraldehyde-3-phosphate dehydrogenase and CuZn superoxide dismutase, and opposite responses between 10-fold and 100-fold light increment for e.g. glutamine synthetase and phosphoglycerate kinase.

**Conclusions:**

The results prove the hypothesis that transcript abundance is poorly linked to *de novo* protein synthesis due to profound regulation at the level of translation. This vertical systems biology approach enables to quantitatively and kinetically link the molecular levels for scrutinizing signal processing and response generation.

## Background

Fluctuating environmental conditions elicit acclimation responses that occur at different molecular levels and on various time scales. For immediate response to light intensity shifts the acclimation includes rapid posttranslational modifications such as reversible protein phosphorylation for state transition or photochemical quenching, and thiol-disulfide transitions of metabolic enzymes [1]. An intermediate response to alter the proteome is mediated by modification of the transcripts recruited to the ribosomes and allows for fast adjustment of *de novo* synthesized proteins [2]. Initiated at the same time scale, transcriptional activity is adjusted, but due to the multiple subsequent steps of transcript accumulation, translation and assembly, the response is somewhat delayed compared to the first and second mechanism [3]. Each level of molecular response is subjected to additional regulation such as RNA stability [4] and dynamics and assembly of complexes [5]. Since these reactions occur outside the organelles for most plastidic proteins their initiation and control depends on retrograde signals from the chloroplast. On a longer time scale reorganization of cell structures, epigenetic control of gene activity and changes in morphology realize additional levels of acclimatory modifications.

This work aims for understanding the different levels of molecular acclimation to high light (H-light). *Arabidopsis thaliana* has been repeatedly used to investigate reactions to H-light. Retrograde signals released within the chloroplast trigger signal transduction pathways that transmit information to the nucleus to modify gene expression necessary for acclimation. The origin and nature of plastid retrograde signals has been deduced from physiological and genetic experiments. Oxidation of the plastoquinone (PQ) pool activates the expression of chlorophyll-a/b-binding protein genes in the nucleus [6]. Redox changes in the intersystem electron transport chain as experimentally induced by preferential excitation of either photosystem II or photosystem I using light quality variation or by addition of inhibitors affect plastid and nuclear gene expression [7]. The thylakoid-associated protein kinases STN7 and STN8 mediate PQ-dependent regulation in the chloroplast, e.g. photosystem II protein phosphorylation, and in the long term response [8]. Signals originating downstream of photosystem I trigger the acclimation responses in chloroplasts and extrachloroplast compartments, e.g. regulating the expression of nuclear encoded 2-Cys peroxiredoxin [9]. Hormonal signals involved in retrograde signalling include abscisic acid, salicylic acid and 12-oxophytodienoic acid, the precursor of jasmonic acid [10–12]. In some cases signalling components such as transcription factors of the AP2/EREBP family participate in the retrograde signalling response. Few involved signalling elements could already tentatively be aligned. Thus, different operational signals from the chloroplast converge upstream of GUN1 and initiate ABI4-dependent nuclear gene expression [13]. Using genetic approaches, singlet oxygen signalling was associated with FLU and the functional executer isoforms EXE1 and EXE2 [14].

The here employed experimental design uses a differential light acclimation regime of *Arabidopsis thaliana* grown at low light near the light compensation point (about 8 μmol quanta^.^m^-2.^s^-1^) and normal growth light (80 μmol quanta^.^m^-2.^s^-1^) with a subsequent transfer to H-light (800 μmol quanta^.^m^-2.^s^-1^) as introduced before [11]. In the previous work transcript, protein and metabolite levels, as wells as activities of components of the water-water cycle were compared with untreated control plants at 6 h and 24 h after the 10- and 100-fold light shift. In addition the response of marker transcripts described as suitable indicators for sugar, ABA, plastoquinone, singlet oxygen, ROS, lipid and overreduction-dependent signalling was determined in that study, suggesting a major signalling function for reductive power, metabolites, and lipids. Strong transcript regulation for water-water cycle enzymes, e.g. stromal and thylakoid-bound ascorbate peroxidases, dehydroascorbate reductase and CuZn-superoxide dismutase, was not reflected at the protein level [11]. This prompted us to ask whether the transcriptional regulation translates into increased protein synthesis.

Few methods allow for analysis of the *de novo* protein synthesis. Among these are the expression of protein fusions with reporters that are detectable *in vivo* or *ex vivo* using endogenous promoters [15], the use of translation inhibitors such as cycloheximide to follow the decline in protein amount and assuming that the difference relative to the non-inhibited conditions represents the contribution of *de novo* protein synthesis [16]. However, the only direct method aims at labelling the *de novo* synthesized protein by incorporation of isotopes which either can be determined mass spectrometrically [17] or in case of radioactive elements can be followed by scintillation counting of immunoprecipitates or autoradiography following 2D separations [18]. Increasing sensitivity, dual labelling methods and quantitative spectral counting in mass spectrometric analysis also give access to *de novo* synthesized proteins if sufficient proportions of the stable isotope are incorporated [17, 19]. At present the sensitivity and broad applicability of radiolabelling to biological samples followed by 2D separation still offers a competitive alternative in a zero background.

Many studies on retrograde signalling from the chloroplast to the nucleus focused on transcript regulation as easy readout and on genetic approaches to identify disturbances. Here we wanted to learn more on retrograde signalling in response to a strong light intensity shift with focus on *de novo* synthesized proteins. Labelling of *de novo* synthesized proteins often coupled to immunoprecipitation has been and is a broadly used method. However, attempts appear to be missing to use this strong technology in the systems biology era. Therefore, we aimed for exploring the potential of using 35S-methionine labelling to assess the coupling between retrograde signalling-induced changes in transcript levels to *de novo* protein synthesis and protein levels.

## Results

Low (L-) and normal (N-) light-acclimated plants were transferred to the same high (H-) light intensity of 800 μmol quanta^.^m^-2^ s^-1^ which is equivalent to a 100- and 10-fold increase over acclimation light, respectively. The experimental design and the response of the plants have been described in detail by Oelze et al. [11]. Table 1 summarizes four parameters measured as basic parameters and taken from Oelze et al. [11]: It can be seen that the L-plants only had 38% of the fresh weight-related RNA of N-plants, 48% protein and 61% chlorophyll. Protein and RNA tented to increase during the H-treatment, however only in the L→H-light treatment protein content increased significantly. Effective quantum yield of photosynthesis decreased significantly during the H-light treatment, albeit less in the N→H-plants than in the L→H-plants. It should be noted that the photoinhibition was entirely reversible [11].

**Table 1:** **Basic characterization of plants grown in normal (N) or in low (L) light, or transferred to high (H) light for 6 h (N→H, L→H)**

Parameter	Treatment			
	N	N→H	L	L→H
**Chlorophyll** [mg/g fw]	1.22 ± 0.10^a^	1.15 ± 0.10^a^	0.75 ± 0.08^b^	0.80 ± 0.10^b^
Φ**PSII** [r.U.]	0.76 ± 0.01^a^	0.62 ± 0.04^c^	0.72 ± 0.02^b^	0.49 ± 0.05^c^
**Protein** [mg/g fw]	10.19 ± 1.01^a^	11.49 ± 0.96^a^	4.94 ± 0.14^c^	5.58 ± 0.33^b^
**RNA** [μg/g fw]	13.92 ± 6.60^a^	17.27 ± 7.91^a^	5.31 ± 3.11^b^	5.89 ± 3.02^b^

Contents of chlorophyll, protein and RNA were determined in leaf samples (n between 3 and 8 independent experiments, m ± SD; different letters mark significance groups according to *t*-test, p ≤ 0.05). Effective quantum yield of photosystem II as measured by pulse amplitude modulated chlorophyll fluorimetry is shown as m ± SD with n = 30 from 3 independent experiments. Letters mark groups of significant difference according to *t*-test, with p ≤ 0.01. Data are from [11].

**Figure 1:**
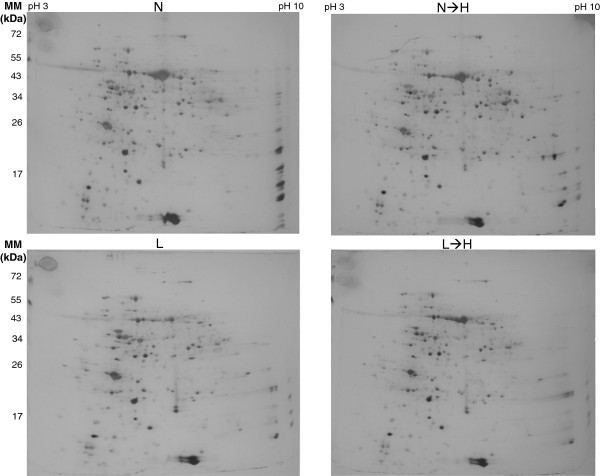
**Two-dimensional electropherograms of leaf proteins from L-, L**→**H-, N- and N**→**H-light plants.** The youngest fully expanded leaves were harvested 6 h after light shift or in the control condition and proteins extracted, and 100 μg of total protein amount was separated as described in M&M. Shown are silver-stained gels representative for three independent experiments.

**Figure 2:**
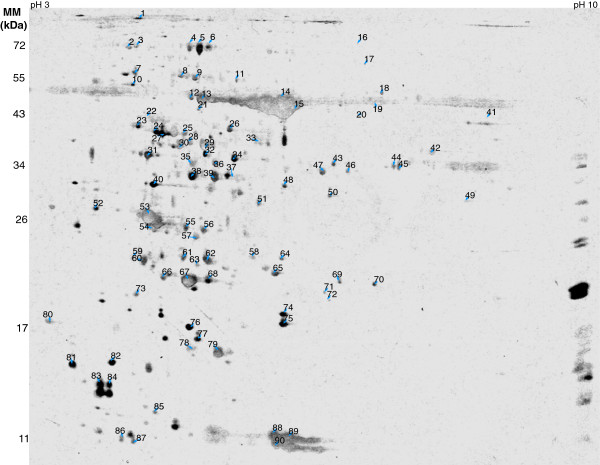
**Annotated reference gel (‘master gel’) for the light shift experiment.** 100 μg of total protein was separated by 2D gel electrophoresis. Spots were excised and 90 polypeptides were identified by mass spectrometric analysis.

H-light triggers the release of retrograde signals which derive from the chloroplast, modify nuclear gene expression and initiate acclimation responses. L- and N-plants revealed 2.219 transcripts with ≥2-fold difference. The transcriptional regulation following transfer to H-light was almost finished after 6 h, with only 205 transcripts remaining differentially expressed between L→H- and N→H-plants [20]. This experimental system has previously been established in order to follow the acclimation process to H-light in particular with focus on the antioxidant defence system after 6 h of H-light exposure [11] and to address involved signalling pathways in a time-resolved manner [20]. The setup appeared suitable to ask the next question concerning the coupling between transcript regulation and *de novo* protein synthesis. To this end leaf proteins were extracted from L-, N-, L→H- and N→H-light samples after 6 h of treatment and subjected to 2D gel electrophoresis with silver staining for sensitive visualization of protein pattern (Figure 1). Polypeptides were excised from parallel gels and subjected to mass spectrometric identification (Table 2). Using this information and 2D analysis software a partially annotated master gel was assembled (Figure 2).

**Table 2:** **Compilation of polypeptides identified both in the silver stained gels and in the autoradiogram**

	Protein name	ATG number	MM (kDa)	Localization	Functional role	Silver	De novo	Mascot score
1.	2-Cys Peroxiredoxin	AT3G11630	22.4	Chloroplast	Defense response	+	+	66
2.	3-Ketoacyl CoA thiolase 3	AT2G33150	48.6	Peroxisome	Fatty acid biosynthesis	+	+	99
3	Ascorbate peroxidase 1	AT1G07890	27.6	Cytosol	Defense response	+	+	253
4.	ATP synthase delta-subunit	AT4G09650	22.8	Chloroplast	ATP synthesis	+	+	260
5.	ATP synthase subunit beta	ATCG00480	47.7	Chloroplast	ATP synthesis	+	+	365
6.	Carbonic anhydrase 1	AT3G01500	25.6	Chloroplast	Carbon utilization	+	+	45
7.	Carbonic anhydrase 2	AT5G14740	25.1	Chloroplast	Carbon utilization	+	+	72
8.	Chaperonin 60 beta	AT1G55490	58.1	Chloroplast	Protein folding	+	+	89
9.	Chloroplast HSP 70-1	AT4G24280	74.6	Chloroplast	Stress response	+	+	528
10.	Chloroplast HSP 70-2	AT5G49910	74.6	Chloroplast	Stress response	+	+	337
11.	Cu/Zn Superoxide dismutase	AT2G28190	15.7	Chloroplast	Defense response	+	+	81
12.	Cyclophilin Cyp 20-3	AT3G62030	19.7	Chloroplast	Rotamase	+	+	207
13.	Dehydroascorbate reductase	AT1G19570	21.7	Cytosolic	Defense response	+	+	93
14.	D-Ribulose-5-P epimerase	AT5G61410	28	Chloroplast	Calvin cycle	+	ND	154
15.	Fructose-bisphosphate aldolase 1	AT2G21330	41.9	Chloroplast	Calvin cycle	+	+	91
16.	Fructose-bisphosphate aldolase 2	AT4G38970	38	Chloroplast	Calvin cycle	+	+	106
17.	GAP C2 subunit	AT1G13440	36.9	Cytosol	Oxidoreductase	+	+	164
18.	Germin 3 oxalate oxidase	AT5G20630	19.5	Apoplast	Defense response	+	ND	243
19.	Glutamine synthetase 2	AT5G35630	42.5	Chloro/Mito	Glutamine biosynthesis	+	+	141
20.	Glutathione S-transferase F8	AT2G47730	23.9	Chloroplast	Stress response	+	ND	71
21.	Glutathione S-transferase F9	AT2G30860	24.2	Cytosol	Stress response	+	ND	118
22.	Glyceraldehyde-3-P-DH, B subunit	AT1G42970	39.3	Chloroplast	Calvin cycle	+	+	70
23.	HCF 136	AT5G23120	38.5	Chloroplast	Photosynthesis	+	+	173
24.	Lactate/malate dehydrogenase	AT1G53240	33.2	Mitochond.	TCA-cycle	+	+	65
25.	Malate dehydrogenase	AT3G47520	34	Chloroplast	Redox metabolism	+	+	107
26.	Manganese SOD	AT3G10920	22.2	Mitochond	Defence response	+	ND	78
27.	O-Acetyl serine thiol lyase B	AT2G43750	35.1	Chloroplast	Cysteine biosynthesis	+	+	85
28.	Phosphoglycerate kinase 1	AT1G79550	42.63	Chloroplast	Calvin cycle	+	+	86
29.	Phosphoglycerate mutase	AT3G08590	60.7	Cytosol	Glycolysis	-+	+	143
30.	Phosphoribulokinase	AT1G32060	39.2	Chloroplast	Calvin cycle	+	+	97
31.	Plastid-lipid-associated protein 1	AT4G04020	34.9	Chloroplast	Stress response	+	+	113
32.	Plastocyanin (DRT 112)	AT1G20340	10.5	Chloroplast	Electron transport	+	ND	169
33.	PSII oxygen evolving complex	AT5G66570	26.5	Chloroplast	Photosynthesis	+	+	114
34.	PSII, subunit PSB-O2	AT3G50820	35.0	Chloroplast	Photosynthesis	+	+	304
35.	PSII subunit P-1	AT1G06680	20.2	Chloroplast	Photosynthesis	+	+	183
36.	Ribose 5-phosphate isomerase	AT3G04790	27.1	Chloroplast	Calvin cycle	+	ND	161
37.	Ribosomal protein S1	AT5G30510	40.5	Chloroplast	RNA binding	+	+	70
38.	RPL12 | ribosomal protein L12-A	AT3G27830	14	Chloroplast	Translation	+	+	78
39.	Rubisco activase	AT2G39730	46.2	Chloroplast	Calvin cycle	+	+	462
40.	RubisCO large subunit	ATCG00490	53	Chloroplast	Calvin cycle	+	+	304
41.	RubisCO small subunit 1A	AT1G67090	14.7	Chloroplast	Calvin cycle	+	+	346
42.	RubisCO small subunit 1B	AT5G38430	14.8	Chloroplast	Calvin cycle	+	+	71
43.	RubisCO small subunit 2B	AT5G38420	14.8	Chloroplast	Calvin cycle	+	+	308
44.	S-Adenosylmethionine synthetase 1	AT1G02500	43.2	Cytoplasm	Met adenos.transferase	+	ND	92
45.	Sedoheptulose-bisphosphatase	AT3G55800	36.1	Chloroplast	Calvin cycle	+	+	229
46.	Stromal APx	AT4G08390	37.8	Chloroplast	Defense response	+	+	67
47.	Thioredoxin m1	AT1G03680	12.4	Chloroplast	Defense response	+	+	105
48.	Thioredoxin m2	AT4G03520	12.5	Chloroplast	Defense response	+	+	72
49.	Triose phosphate isomerase	AT2G21170	27	Chloroplast	Calvin cycle	+	+	133

Shown are the specific details about size, predicted localization, the functional role of the proteins and the MASCOT score. MG #: number in annotated master gel; +: Unequivocally identified by mass spectrometry with at least two peptides; +-: tentatively identified by one peptide; ND: not detected. Polypeptides #5 and 40 are plastome-encoded.

ND- not detected in autoradiograms.

**Figure 3:**
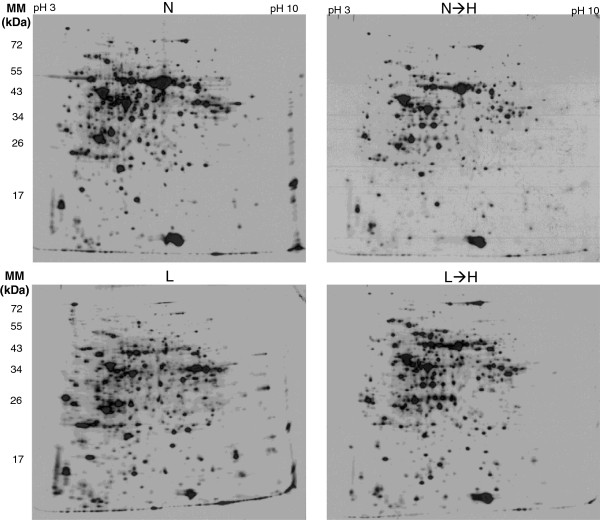
**Two-dimensional autoradiograms of** ***de novo*** **-synthesized proteins in leaves from L-, L**→**H-, N- and N**→**H-light treated plants.** ^35^S-methionine was applied to the leaf surface at t = 4 h after transfer to H-light and the controls. Leaves were harvested at t = 6 h. Samples equivalent to 10^6^ counts per minute were loaded on each gel. The gels were prepared for autoradiography and x-ray films exposed for 48 h at -80°C. The experiments were conducted three times and representative autoradiograms are shown.

**Figure 4:**
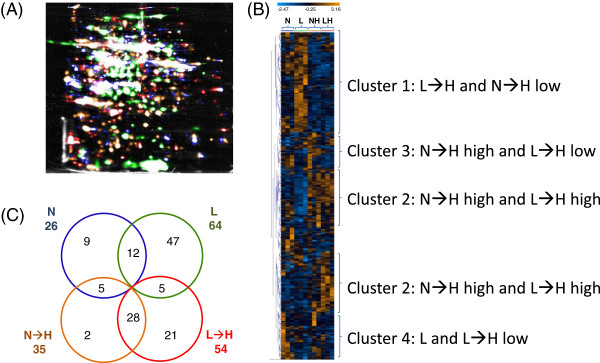
**Analysis of the autoradiograms for changes in reliably detected** ***de novo*** **synthesized proteins.** Three autoradiograms for each condition from independent experiments were analysed with the Delta 2D software. **(A)** The results from three gels were fused and spots color-coded: N = blue, N→H = orange, L = green, L→H = red. **(B)** A heat map was automatically constructed as described above based on the set of 129 reliably detected changes that were classified as significant with one way ANOVA (p ≤ 0.01). The lanes of the three identical conditions were placed next to each other. The four clusters were categorized according to the automatically generated cluster tree depicted on the left hand side. **(C)** Venn diagram of the significantly up-regulated spot intensities representing the overlaps among treatments.

In the next step, radioactively labelled 35S-methionine was applied to leaf surfaces of intact plants in the identical experimental setup at 1 pm, i.e. 4 h after the beginning of H-treatment, and the plants were further incubated for two more hours. Thus, harvesting and analysis of *de novo* synthesized proteins occurred 6 h after transfer to H-light. For analysis protein extracts of labelled leaves equivalent to 10^6^ counts per minute were subjected to 2D PAGE and analysed by autoradiography (Figure 3). The four conditions resembled each other in the basic pattern of a large set of proteins, but also revealed significant differences, particularly between L- and L→H-plants on the one hand and N- and N→H-plants on the other. The most obvious difference was monitored for RubisCO large subunit which was synthesized both in N- and N→H-plants at high rates, but label was almost absent in L-plants and only slightly induced in L-plants upon transfer to H-light. All gels from the three independent experiments were matched to generate a fused master gel image utilizing Delta 2D software (Figure 4) and analyzed for spot response behaviour. In total 129 spots could be identified that revealed differences among the treatments with statistical significance <0.01 (one way ANOVA). The clustered heat map for three experiments with 12 samples and 129 significantly altered spots is depicted in Figure 4B. It shows (a) a consistent regulation for same treatments in the three independent experiments, (b) the contrasting regulatory state of L-plants compared to that of all other treatments, and (c) the efficiency of L→H-plants in adjusting the pattern of *de novo* synthesized proteins to that of N→H-plants despite the different starting points. Four major cluster types of regulation could be identified: Polypeptides of cluster 1 were synthesized at low *de novo* rates in N→H- and L→H-plants, polypeptides of cluster 2 were high in N→H- and L→H-plants. Cluster 3 includes polypeptides whose synthesis showed contrasting responses in H-light, i.e. stimulation in N→H and low synthesis in L→H-plants, while cluster 4 showed the opposite. Focussing on proteins being synthesized above (‘up-regulated’) or below average allowed the generation of a Venn-diagram (Figure 4C), that confirmed the impression from the heat map, namely that the labelling pattern of N-plants was most closely related to the average state with only 26 spots (20%) synthesized above or below average of all treatments, 9 of which were specific to N-plant, 12 overlapped with L-plants and 5 with N→H-plants. Radiolabel of 50% (=64) of the spots in L-plants deviated from average; 47 being specific and only 5 were present in a distinct amount after transfer to H-light. Levels in 22% (28) spots deviated from average in N→H- and L→H-plants.

Autoradiographs were digitalized and the spot landscape warped to the master gel image. The protein pattern of *de novo* synthesized and, thus, radiolabelled polypeptides differed considerably from silver- or Coomassie-stained 2D patterns (Figures 1, 2 and 3). Despite these differences, both patterns could reliably be matched since many spots served as unambiguous landmarks. In the next step all 12 gels from four conditions with three experiments were matched, the spot volume as well the greyness quantified and the annotated polypeptides were confirmed manually. The results of these 49 polypeptides are given in Table 2 which lists the AT number, molecular mass, localization, detection in the silver stained gels or autoradiograms and the MASCOT score. Eight polypeptides detected in the annotated gels were not found in the autoradiograms. The vast majority of polypeptides, namely 80% showed a proven or predicted chloroplast localisation. *De novo* protein synthesis of these proteins was investigated for its response to the treatment and assigned to the four major response clusters (Table 3). In cluster 1 “change in *de novo* protein synthesis down in both H-treatments” appeared polypeptides with function in photosynthetic electron transport and antioxidant defence. Chaperones and proteins of redox homeostasis were found in cluster 2 “up in both H-treatment”. Metabolic enzymes predominated cluster 3 “up in N→H and down in L→H-plants”, and cluster 4 “N and N→H low” with ascorbate peroxidases and redox regulatory elements such as cyclophilin Cyp20-3 and malate dehydrogenase.

**Table 3:** **Clustering of** ***de novo*** **synthesized proteins with identified functional assignment**

Cluster	Response pattern	Transcripts/genes	Functional role
**1**	**L**→**H & N**→**H low**	DHAR	Antioxidant defence
		GAPDH B subunit	Photosynthesis
		PSII subunit O-2	Photosynthesis
		PSII subunit P-1	Photosynthesis
		Ribose 5-P isomerase A	Photosynthesis
		RPL12, ribosomal protein	Protein synthesis
		SOD, Cu/Zn	Antioxidant defence
		Thioredoxin m2	Redox regulation
**2**	**L**→**H & N**→**H high**	Carbonic anhydrase 2	Photosynthesis
		Chaperonin 60 beta	Protein folding
		FBP aldolase1	Photosynthesis
		FBP aldolase 2	Photosynthesis
		HCF 136	Photosynthesis
		HSP 70-1, cp	Stress response
		Lactate/malate DH	Respiration
		O-Acetyl serine thiol lyase B	Sulfur metabolism
**3**	**N**→**H high, L**→**H low**	3-Ketoacyl CoA thiolase 3	Fatty acid metabolism
		GAP C2 subunit	Photosynthesis
		Glutamine synthetase 2	Nitrogen metabolism
		Phosphoglycerate kinase 1	Photosynthesis
		Phosphoribulo kinase	Photosynthesis
		Rubisco activase	Photosynthesis
		Plastid-lipid-associated protein 1	Stress response
		RubisCO SU 1A	Photosynthesis
		SBPase	Photosynthesis
		SAM synthetase 1	Sulfur metabolism
**4**	**N, N**→**H-high; L, L**→**H-low**	ATP synthase beta	Photosynthesis
		ATP synthase delta	Photosynthesis
		APX 1	Antioxidant defence
		APx, stromal, cp	Antioxidant defence
		Carbonic anhydrase 1	Photosynthesis
		Cyclophilin Cyp 20-3	Redox regulation
		Malate DH cyt	Redox regulation
		PSII OEC	Photosynthesis
**No peculiar group pattern**		2-Cys Peroxiredoxin	Antioxidant defence
		Germin 3 oxalate oxidase	Stress defence
		GST F8	Stress defence
		GST F9	Stress defence
		HSP 70-2, cp	Stress defence
		Malate DH, cp	Redox regulation
		Mn SOD	Antioxidant defence
		Phosphoglycerate mutase	Glycolysis
		Plastocyanin (DRT 112)	Photosynthesis
		Ribosomal protein S1	Protein synthesis
		RubisCo large subunit	Photosynthesis
		Ribulose-5-P epimerase	Photosynthesis
		Thioredoxin m1	Redox regulation
		Triosephosphate isomerase	Photosynthesis

The proteins were clustered using the Delta 2D-software package and assigned to four major types of regulation (Clusters 1 to 4) as outlined.

**Figure 5:**
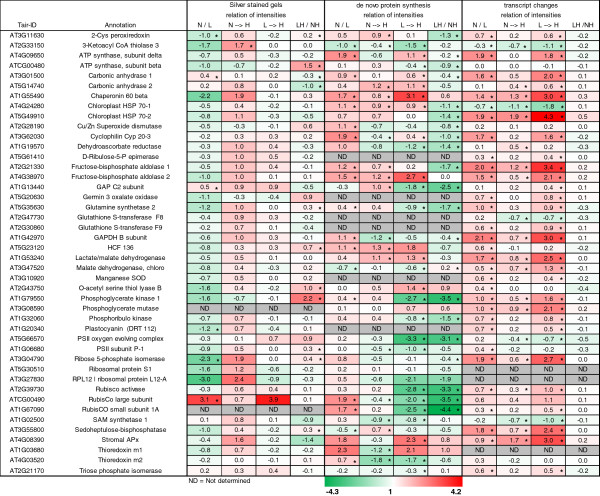
**Comparison of light-dependent changes in spot intensity in silver stained gels, autoradiograms and in transcript levels.** Changes in spot intensities of silver gels and autoradiograms were taken from the three independent experiments similar to Figures 1, 2 and 3. Transcript data were extracted from three independent sets of array hybridisation [20]. Changes calculated as value at higher light intensity divided by intensity at lower light intensity were colour-coded as indicated in the colour bar at the bottom (asterisks indicate significant difference of changes, *t*-test (p < 0.1 for *de novo* synthesis, p < 0.05 for transcript).

**Figure 6:**
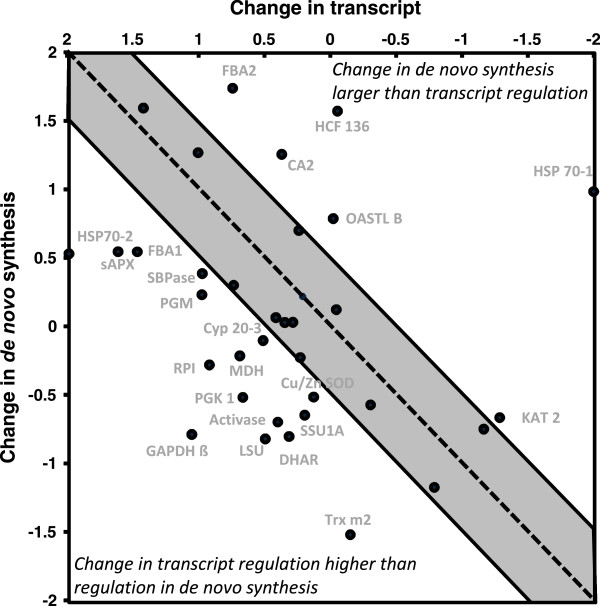
**Correlation of transcript regulation with regulation of** ***de novo*** **synthesized polypeptides.** The figure combines the results from both experiments the L→H- and N→H-light transfer. The log_2_-fold change of each transcript and radiolabelled protein, respectively, was divided by the highest value of regulation observed. The sum of both numbers obtained for *de novo* synthesis was plotted against the sum of both numbers for transcript regulation. A value of 2 denotes maximal up-regulation, a value of -2 maximal down-regulation in both treatments. The shaded area covers all values with regulation below or equal to 0.5-fold up or 0.5-fold down. All spots outside the shaded area show deviation between transcript regulation and de novo protein synthesis. Abbreviations: CA: carbonic anhydrase; Cyp20-3: cyclophilin 20-3; DHAR: dehydroascorbate reductase; FBA: fructosebisphosphate aldolase; GAPDH: glyceraldehyde-3-phosphate dehydrogenase; KAT: ketoacyl CoA-thiolase; LSU: RubisCO large subunit; MDH: malate dehydrogenase; OASTL B: O-acteylserine thiol lyase; PGK: phosphoglycerate kinase; PGM: phosphoglycerate mutase; RPI: ribose-5-phosphate isomerase; SBPase: sedoheptulose-1,7-bisphosphatase; SSU: RubisCO small subunit; Trx m2: thioredoxin m2.

As reported before, RNA was isolated from leaves treated as above (L-, N-, L→H, N→H) at t = 6 h. ATH1 whole genome arrays were hybridized from three experiments [20]. Raw data were processed with ROBIN (MPI Golm, Germany) and normalized on total intensity of all spots (RMA normalisation [21]. Means and corrected standard error (p < 0.005) were calculated [22, 23]. Transcripts identified in the autoradiograms and silver stained gels were selected from the list of transcripts and ratios of change were calculated. Figure 5 summarizes the results for the protein, *de novo*-synthesized and transcript level by heat map representation. Total protein was unrelated to transcript levels and *de novo* protein synthesis rates. However, also changes in transcript levels were unrelated to *de novo* synthesis for most genes. The changes upon the 10- and 100-fold light shift in transcript amounts were related to the changes in *de novo* protein synthesis and plotted in a diagram (Figure 6).

## Discussion

### Reorganization of the leaf proteome in light acclimation

Sun and shade acclimation depends on structural and functional reorganization of photosynthetic organs [24, 25]. Total leaf protein amount related to fresh weight differed between plants grown under L- or N-light conditions more than twofold. Two possible reasons might exist, namely either a similar protein complement at lower level or a profound qualitative difference that explains the lower level. Since plasmatic compartments such as cytosol, matrix and stroma contain about 25% (w/v) protein, e.g. 10 mg protein/40 μl chloroplast volume [26, 27], a twofold difference clearly indicates that the volumes of plasmatic compartments is strongly decreased after the 10 d L-light acclimation [11]. But in addition to a general decrease in volume, polypeptide composition also changes qualitatively. The best established example of light acclimation-dependent differences in protein composition concerns the increase in D1 protein and the decrease in light harvesting complex proteins (LHCII) with increasing growth light [28]. Changes in the photosynthetic apparatus are instrumental to adjust energy conversion and growth and are also important for optimized resource allocation, e.g. in dependence on light and nitrogen availability [29]. Protein patterns of silver-stained electropherograms differed between L- and N-acclimated plants. Many polypeptides appeared to be less abundant in N-light plants than in L-plants. This may be explained by normalization of each spot on total intensities in the gels. Due to the high RubisCO amount in extracts from N-plants, the intensities of most other bands will appear to be lower. But considering the low fresh weight-related protein contents of L-plants it becomes clear that the polypeptide abundance in silver gels would need some correction if polypeptide abundance should be related to fresh weight. Abundance of only few proteins changed during the 6 h period of H-light treatment. RubisCO was among the significantly accumulating proteins in the L→H-plants. It should be noted that the combined evaluation of both light shift treatments appeared justified despite in some cases different starting points due to the mostly similar response of protein abundance (82% similar response) and transcript regulation (100% similar response). This regulation leads to a highly similar transcriptome state after 6 h H-light [20].

### Strengths and drawbacks of *in vivo* labelling of *de novo* synthesized proteins

Acclimation responses to environmental conditions are most frequently analysed at the level of specific transcripts or of genome-wide transcriptomes [30]. The matching of annotated silver-stained or Coomassie-stained 2D gels with autoradiograms was expected to allow for protein assignments of *de novo* synthesized polypeptides. But the labelling method needs some discussion. Labelling of intact plant tissue with ^35^S-methionine requires time for uptake and incorporation, and in some studies it was achieved by wounding [31], in others by feeding via the transpiration stream [18] or by application to tissue surfaces. We chose the application to the cuticular surface of the youngest fully expanded leaves because neither application to the transpiration stream e.g. by injection or wounding, appeared suitable for our purpose of undisturbed but sensitive labelling of newly synthesized proteins. Labelling de novo synthesized leaf proteins by feeding the labelled amino acid to roots unlikely would allow for sufficiently strong incorporation within 2 h, but this could be compared in the future. The experimental design required incubation time for sufficient incorporation. Nevertheless, radiolabelling still is the only method at hand that allows for rapid, sensitive and reliable labelling of the *de novo* synthesized protein. It may be expected that with further advancement of mass spectrometric analysis, stable isotopes will offer alternative methods to study protein turnover also for eukaryotic multicellular organisms similar to unicellular organisms that can easily be labelled in suspension [32]. A recent review summarizes the strategies to label *de novo* synthesized proteins by modern proteomics [33]. The here employed method should be added to the portfolio of potential options that can be employed. Starting 4 h after transfer to H-light appeared suitable because many transcriptional changes had been shown to reach a new steady state at this time, e.g. sAPX [11] or monodehydroascorbate reductase, ABA-dependent cold regulated 47 (COR47), pyruvate kinase related protein (PKRP) [20]. Thus, the labelling that starts after translocation of ^35^S-methionine through the cuticle to the mesophyll reflects a transcriptional state similar to 6 h after transfer to H-light for which the transcript analysis has been performed.

### Apparent absence of coupling between transcript regulation and *de novo* protein synthesis

The comparison of transcript regulation with differences in *de novo* synthesized protein demonstrates the flexible coupling between transcript regulation and translation (Figure 6). Piques et al. [34] compared transcript levels, ribosome occupancy, enzyme protein amount and activity at different times of day. Their scatter analysis revealed a poor dependency of ribosome loading on total amount of investigated transcripts. The Pearson’s correlation coefficient was 0.065 in the dark period and 0.102 in the light period [34]. Here, transcript analysis revealed efficient regulation following transfer to H-light. In sum 27 out of 42 transcripts of identified proteins, i.e. 64%, had log_2_-fold differences ≥|0.5| between N- and L-light grown plants prior to H-light treatment. The size of this group of differentially regulated transcripts decreased to only 2 genes after 6 h of H-light. Thus, transcript regulation within this selected set of identified proteins was entirely in line with the global regulation of the transcriptome after 6 h of H-light [20]. Thus transcriptional regulation in response to H-light was almost completed after 6 h H-light.

In most cases regulation of transcript amounts was more pronounced than regulation of *de novo* protein synthesis. Regulation of 6 proteins occurred much stronger at the level of *de novo* protein synthesis. Several translation factors have been identified as target of posttranslational regulation including thiol-disulfide transitions [35], glutathionylation [36], phosphorylation [37] and S-nitrosylation [38]. Among the targets researchers identified several ribosomal proteins (RPL S1, S6, L13, L30), elongation factors (EF-Tu, EF-G, EF-2, EF-1α) and enzymes such as nucleoside diphosphate kinase III and tRNA synthetases which all are involved in translation. Redox changes, ROS production and activation of phosphorylation cascades have been implicated in retrograde signalling. The protein kinases STN7 and STN8 mediate light-dependent reorganization of the photosynthetic apparatus [39]. ROS waves adjust nuclear gene expression in excess light acclimation [40]. ROS and redox feed into the mitogen activated protein kinase pathway [41]. Translational activity is strongly altered by ROS in yeast [42]. Thus, translation in plants is a prime but hitherto not sufficiently explored target of retrograde signalling as underlined by the data presented in this paper. The reader is also referred to the metaanalysis by Schwarzländer et al. [43] who observed that transcripts encoding for proteins involved in protein synthesis are significantly affected by retrograde signals released from the mitochondrion.

### Functional implications of translational control of identified targets

Control of posttranscriptional processes accelerates the speed and versatility of stress acclimation. The high significance of specific transcript recruitment to ribosomes in plants has best been demonstrated for acclimation to hypoxia [44]. The authors showed hypoxia-specific changes of transcriptome and translatome at the global, organ- and cell-specific level. Preferential ribosome association was observed for sucrose transporters, heat shock factors and transcription factors [45]. Here, expression of six genes was more strongly regulated at the level of protein synthesis than of transcript accumulation. It may be assumed that the gene product functions are needed after transfer to H-light. Despite down-regulation at the transcript level, ^35^S-methionine incorporation into HSP70-1 still occurred at high rates. In a converse manner, HSP70-2 was synthesized at similar rates despite a large increase in transcript amount. Chloroplast HSP70s facilitate protein import into the chloroplasts, a function which is of eminent importance during environmental transition such as exposure to excess excitation energy [45]. High chlorophyll fluorescence HCF136 was identified in a screen for genes with function in assembly of functional photosystem II [46]. FBP aldolase as part of the Calvin cycle, O-acetyl serine thiol lyase with its function in cysteine synthesis, carbonic anhydrase which facilitates equilibration between carbonate and CO_2_ as substrate of the Calvin cycle and 3-ketoacyl CoA thiolase 3 involved in fatty acid synthesis showed stimulated *de novo* synthesis. This type of regulation may easily be reconciled with their metabolic functions which are important for H-light acclimation. Arguments appear less straight forward when it comes to explain the low level of *de novo* protein synthesis observed for 16 genes. They mostly function in metabolism such as seduheptulose-1,7-bisphosphatase which is suggested to limit Calvin cycle activity [47], large and small subunits of RubisCO, RubisCO activase, phosphoglycerate mutase, phosphoglycerate kinase and ribose-5-phosphate isomerase. Others are involved in redox homeostasis and antioxidant defence (malate dehydrogenase, dehydroascorbate reductase, superoxide dismutase, stromal ascorbate peroxidase and the regulator of chloroplast cysteine synthase complex cyclophilin Cyp20-3 [12]. It may be hypothesized that these proteins are present at sufficient amounts prior to H-light treatment and that the low ratio of *de novo* synthesis-to-transcript amount merely reflects such mechanisms of yet un-understood feedback control. It should be noted that photoreceptor-dependent signaling might contribute to the transcriptional and translational responses described in this paper, albeit previous work largely excluded a major role of photoreceptors in this particular experimental setup [7, 11].

## Conclusions

Translational control is still poorly investigated particularly in plants: Initiation, elongation and pausing contribute to transcript selection and efficiency of translation. *De novo* labelling as used here determines the outcome of all these processes and, therefore is a better readout of protein synthesis than ribosome loading eventually combined with ribosome footprinting [48]. The latter technique allows for profiling of RNA sequences by deep sequencing that are protected from degradation by associated ribosomes. Our study adds a novel method to the portfolio available to investigate posttranscriptional regulation. The results show that H-light acclimation involves translational control as decisive part of retrograde signalling and concerns a large fraction, namely almost 2/3 in the set of identified proteins. Furthermore the rate of *de novo* protein synthesis cannot directly be predicted from transcript levels.

## Methods

### Plant growth and treatment

*Arabidopsis thaliana* was grown in a growth chamber in a mix of 50% soil, 25% Perlite and 25% Vermiculite, supplemented with one dose of Lizetan (Bayer, Germany). Following seed stratification for 2 d at 4°C, plants were grown for 30 d in 80 μmol quanta^.^s^-1.^m^-2^ (N-light) with a 14 h light and 10 h dark phase. Subsequently, plants were transferred to 8 μmol^.^s^-1.^m^-2^ (L-light) for 10 d prior to the experiment with transfer to 800 μmol^.^s^-1.^m^-2^ (H-light; 100-fold light increase). The L-plants have been shown to be entirely shade acclimated [11]. Another set of plants was grown in N-light for the whole period of 40 d and also transferred to 800 μmol^.^s^-1.^m^-2^ (10-fold light increase). Control plants were kept in L- and N-light, respectively, and harvested in parallel to the H-light rosettes. Harvest time was always at 3 pm. Chlorophyll, protein and RNA contents and effective quantum yield of photosystem II by pulse amplitude modulation (PAM) were determined as described in Oelze et al. [11].

### In vivo labelling of de novo synthesized proteins

L-[^35^S]-methionine (NEG009T, Perkin Elmer, MA, USA) was supplemented with 0.1% (v/v) Triton X-100 and applied to leaf surfaces with a radioactivity of 20 μCi per leaf. For each treatment 20 μCi were administered to fully expanded leaves from three different rosettes 4 h after transfer to H-light. After 6 h, the leaves were excised from the rosettes, washed first with 0.1% (v/v) Triton X-100 and then with 0.5 mol/L Tris-Cl, pH 6.8.

### 2D-gel electrophoresis

Leaves were ground with a pestle in 1 mL acetone/trichloroacetic acid/β-mercaptoethanol (89.93:10:0.07% v/v) according to Méchin et al. [49]. Following precipitation at -20°C for at least 1 h and subsequent centrifugation, the pellet was washed and sedimented thrice with ice-cold acetone/β-mercaptoethanol, dried and resuspended in lysis buffer [50]. For radioactive samples, incorporated ^35^S was quantified by precipitating aliquots on Whatman filter followed by scintillation counting. For silver-stained gels, protein amounts were quantified at 595 nm with the BioRad protein assay. Separation in the first dimension was achieved with Immobiline™ DryStrips (pH range 3-10 NL, 18 cm, GE Healthcare, Uppsala, Sweden). Sample equivalent to 100 μg protein or 10^6^ cpm was dissolved in 340 μL complete rehydration buffer (8 mol/L urea, 2% (w/v) CHAPS, 0.002 bromophenolblue, 0.3% ampholyte, 1.4% (w/v) dithiothreitol) and applied to the Immobiline strips. The rehydration and isoelectric focusing protocol consisted of the steps as follows: 1 h 0 V, 12 h 30 V, 2 h 60 V, 1 h 500 V, 1 h 1000 V, 1000-8000 V for variable time to reach 42000 Vh. Separation in the second dimension was performed on a 12% (w/v) SDS-PAGE of 18 cm length at 40 mA. Silver staining was performed according to Blum et al. [51] and autoradiography as described in Dietz and Bogorad [52].

### Analysis of 2D-gels and heat map construction

Delta 2D software (Decodon, Greifswald, Germany) with its SmartVectors Technology was used to align the gel images to each other to allow for efficient and reliable spot matching. A fusion image was generated containing all spot positions. Each gel was matched with this master gel. Spot boundary detection, pixel intensity quantification and statistical analysis (one way ANOVA) were performed with the built in TIGR MeV tool. Before constructing the heat map, the data set was standardized to zero mean and unit variance. Clustering was achieved using the eucledian distance and complete linkage- default settings of the delta 2D software (DECODON, Greifswald, Germany).

### ATH1-genome array hybridisation and analysis

Isolated total RNA was sent to KFB-company (Competence Centre for Fluorescence Bioanalytics, Regensburg, Germany), processed, and derived fluorescent probes hybridized against the 25mer oligonucleotide ATH1-genome array (Affimetrix, Santa Clara, USA). Glyceraldehyde-3-phosphate dehydrogenase, actin and ubiquitin were used as reference transcripts. The raw data were fed into ROBIN (MPI Golm, Germany). Statistical evaluation of the data was based on the corrected p-value [22, 23].

### Protein identification by mass spectrometry

Corresponding areas of interest were excised from the 2D gels and washed with (a) two times a solution containing trifluoroacetic acid (0.1% w/v) and acetonitrile (60% v/v), (b) acetonitrile (50%), (c) acetonitrile (50%)/50 mM NH_4_HCO_3_ for 0.5 h, and (d) acetonitrile (50%)/10 mM NH_4_HCO_3_ at 21°C for 0.5 h each. Dried gel slices were resuspended in trypsin solution (0.013 mg sequencing quality trypsin (Promega, Mannheim, Germany) in 10 mM NH_4_HCO_3_ pH 8.0) at 4°C for 0.5 h and afterwards at 37°C for about 15 h. Digestion solutions were supplemented with cyano-4-hydroxy-cinnamic acid at a 60:40% ratio. Mass spectra were determined using a Biflex III matrix-assisted laser desorption/ionisation-time of flight mass spectrometer (MALDI-TOF)-MS (Bruker, Bremen, Germany) (previously described [53]). The peptide mass fingerprints (PMF) obtained by tryptic digested proteins were analyzed by MALDI-TOF-MS and proteins were identified by MASCOT (Multiple-Access Space-Time Coding Testbed) software and the National Center for Biotechnology Information (NCBI) protein database. The program compares the peptide masses obtained from experimental digestion to the predicted peptide masses from the theoretical digestion of proteins.

### Correlation of *de novo* protein synthesis and transcript regulation during H-light treatment

The obtained values of the spot intensities for the autoradiograms by Delta 2D were used to calculate the ratios between the different treatments (N/L, N→H, L→H, L→H/N→H). The ratios were recalculated as log_2_-fold change values, to be easily comparable to the obtained log_2_-fold change values of the microarray experiments by ROBIN.

For the comparison of *de novo* protein synthesis and transcriptional regulation the maximum reactions (up or down regulation) for both H-light treatments (L→H, N→H) were used as reference. Each value (F_POI_) of the different targets was divided by the appropriate maximum reaction (F_Ext_; up-regulation was divided by maximum positive reaction while down regulated targets were divided by the maximal negative reaction) for each treatment (N→H or L→H) and for both methods (*de novo* protein synthesis or transcript regulation). Afterwards the calculated values for both *de novo* protein synthesis reactions (N→H or L→H light shift) or for both transcriptional regulations were summed up to give the response factor R.

Therefore, the maxima of regulation would fit in the range between -2 and 2. To evaluate the relationship between *de novo* synthesis and transcriptional regulation, the calculated values were plotted in a diagram where deviation from the diagonal ≤0.5 was set as a cutoff (gray shaded area) and only larger deviations (outside this area) were accepted to indicate distinct regulation between transcript and de novo protein synthesis.

## Abbreviations

2D: Two dimensional
ABA: Abscisic acid
ABI: ABA insensitive
GUN: Genome uncoupled
H: High light
L: Low light
MS: Mass spectrometry
N: Normal light
PAGE: Polyacrylamide gel electrophoresis
PQ: Plastoquinone
ROS: Reactive oxygen species
RubisCO: Ribulose-1,5-bisphosphate carboxylase oxygenase.
